# Potential barriers in implementing the rural toilet retrofitting project: A qualitative study in Jiaozuo, China

**DOI:** 10.3389/fpubh.2022.965150

**Published:** 2022-09-06

**Authors:** Yuan Wang, Yueqi Zhu, Caiyun Qi, Lu Li

**Affiliations:** ^1^Department of Labor and Social Security, School of Philosophy and Sociology, Jilin University, Changchun, China; ^2^Department of Social Work, School of Philosophy and Social Development, Shandong University, Jinan, China

**Keywords:** rural toilet retrofitting, sanitation, Van Meter and Van Horn policy implementation approach, implementation barriers, China

## Abstract

**Background:**

China has launched a large “toilet revolution” in rural areas, but the results show that sanitation has not always been markedly improved. Few scholars have paid attention to this issue, and the list of the reasons is scattered and incomplete.

**Method:**

Using the qualitative research method, this study interviewed seven village cadres and 39 villagers in three villages of Jiaozuo City to examine the implementation barriers to rural toilet retrofitting (RTR) projects in China.

**Results:**

Using the Van Meter and Van Horn policy implementation approach, the research has found that: (a) unreasonable standards and objectives fail to incorporate local conditions and improve the actual quality; (b) lack of capital and human resources cannot renovate new toilets; (c) uncoordinated inter-organizational communication and enforcement activities by top-down mechanism lead to policy implementers and target groups' dissatisfaction with the policy; (d) weak and unenthusiastic, inadequate implementing agencies, reduce their working ability; (e) inappropriate economic, social and political conditions impede the villagers' acceptance; and (f) an attitude of passive acceptance by the implementers reduce the working motivation.

**Conclusion:**

To improve sanitation in rural China, it is necessary to solve the six barriers. The findings of this study can provide recommendations and guidance for implementing the RTR and related public health policies.

## Introduction

Sanitation facilities are directly related to the public's health, and that connection has aroused great concern worldwide. The UNICEF and the World Bank estimated that 616 million people worldwide still use unimproved facilities in 2020. Of these, 494 million were still forced to practice open defecation—- at least nine out of 10 people living in rural areas (1). Inadequacy of sanitation is a significant cause of environmental pollution (2–4). Moreover, fecal and urine contain a variety of pathogens, posing a severe risk to public health (5, 6). Research has shown that 1.8 million people die from diarrheal diseases (including cholera), 88% of which are attributed to an unsafe water supply, inadequate sanitation, and poor hygiene each year (7). Direct global economic losses from these poor sanitary conditions amount to USD 260 billion per year (8).

China's poor sanitation is also extremely serious, with poor toilet sanitation causing serious health problems in ~17 million households in China every year (9). Traditional toilets also cause severe environmental pollution in rural areas, with significantly higher nitrogen emissions than in urban areas (10), and roughly 50 percent of rural water sources are polluted (11). Therefore, improvements in toilet conditions and hygienic habits are critical—- in terms of gastrointestinal diseases alone, the promotion of sanitary conditions can reduce the incidence of diarrhea by 32 percent, and improved sanitation interventions can reduce cases of diarrhea by 45 percent (12).

In 2010, the United Nations General Assembly (UNGA) explicitly recognized access to improved sanitation services as a fundamental human right. As a global goal, many countries have implemented a series of policies and programs to increase the number of sanitary toilets and improve health infrastructure in rural areas. The Chinese government also attaches great importance to rural toilet retrofitting (RTR) projects. In February 2018, the General Offices of the CPC Central Committee and the State Council issued the *Three-year Action Plan for Improving the Rural Living Environment*, which explicitly addressed the treatment of the RTR. From then on, China has been conducting the largest-scale RTR in the world (13), and according to related data statistics and literature, China has achieved remarkable results in its RTR (14, 15).

Despite the infrastructure upgrading driven by China's RTR, only 25% of the households were satisfied with their sanitation system (16). And, by conducting in-depth research on China's RTR, we were surprised to find that not many households are really using the harmless renovated toilets^1^. First, many households still have not undergone toilet retrofitting. In addition, even among those who have, many do not use the renovated toilet and instead choose to continue using a dry latrine or open defecation. Why does a large number of people ultimately reject a good project?

The answer to this question requires an understanding of the factors that may prevent the implementation of RTR. Previous studies have noticed that successful implementation of the RTR in China is hindered by several factors, mainly geographic and technological constraints (17, 18), and funding barriers (9, 19, 20). From the cultural perspective, previous studies have focused on the life concept, sanitation knowledge, and religious beliefs that affect villagers' willingness to participate (21, 22). And the positive willingness of local villagers to pay and participate will promote rural sanitation facilities (23, 24). RTR is a continuous process that includes the design, construction and operation phase (25). In contrast, these studies focused on the barriers in the construction phase of RTR and ignored the problems of low utilization during the operation phase. The use of retrofitted toilets effectively reduced the incidence of hepatitis A and dysentery (26). Only the sustainable use of sanitation facilities will achieve the policy objectives and improve the villagers' health. Therefore, to improve villagers' health, we need to pay more attention to the obstacles of the operation phase. In addition, Jiang pointed out that the RTR face institutional obstacles, such as restrictions from the government management system, lack of subject consciousness, and insufficient social participation (27), adding a political approach and trying to answer the questions related to the two phenomena. However, the single perspective neglects some essential factors, thus failing to provide a comprehensive, systematic and in-depth explanation.

### Conceptual framework

**Figure 1:**
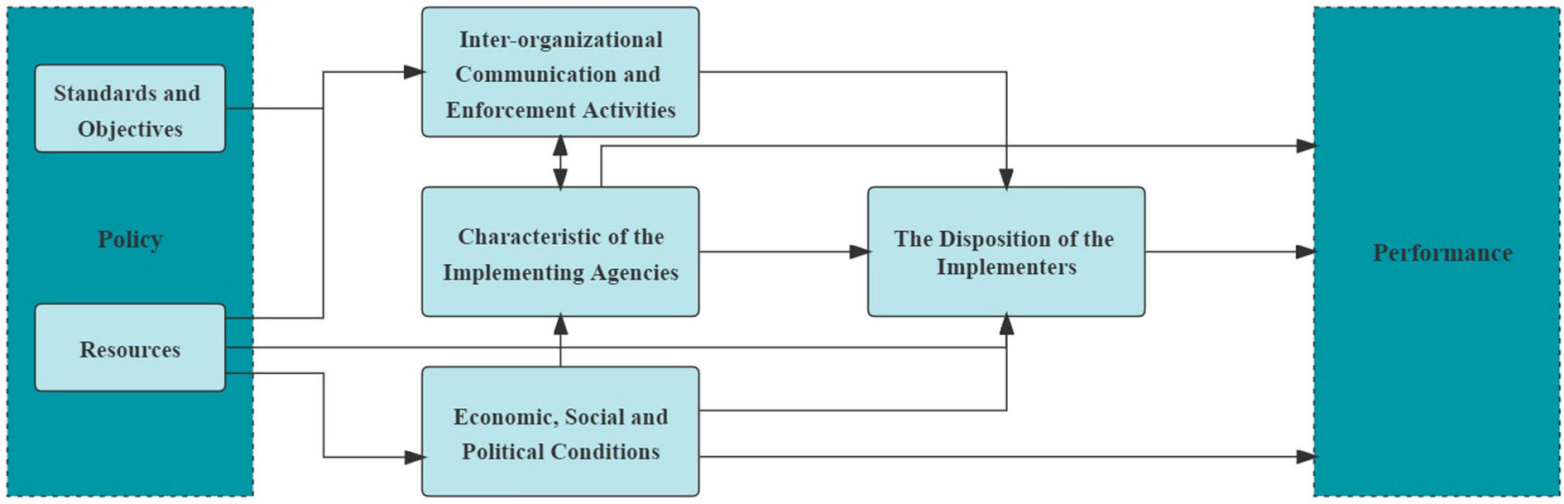
The Van Meter and Van Horn top-down policy implementation approach.

In order to further explore the multidimensional factors causing the failure of the RTR to achieve the expected goals, we sorted out those factors by using the Van Meter and Van Horn policy implementation framework. Donald Van Meter and Carl Van Horn proposed in 1975 that the success or the degree of success of policy implementation is determined by six variables (see Figure 1).

(a) Policy standards and objectives are first. These standards and objectives also provide a basis for policy performance evaluation.
(b) Policy resources, including available and manageable information resources, human resources, authority resources, financial resources, and so forth, are second.
(c) Inter-organizational communication and enforcement activities, which refer to how policymakers, policy implementers, and target groups interact, such as through communication, coordination, and coercion, are third.
(d) Characteristics of the implementing agencies are fourth. This group comprises the characteristics of formal and informal organizations, such as the agencies' organizational level, degree of activity, and personnel configurations.
(e) The system environment of policy implementation, comprising primarily the geographical, political, economic, and sociocultural environments, are fifth.
(f) Finally, sixth is the disposition of the implementers—-their cognition, comprehension, and understanding of the policy, the direction of their response to the policy, and the intensity of their response (28).

The Van Meter and Van Horn policy implementation approach focuses on six key factors affecting policy implementation, including technological, environmental, cultural, political and other variables, and classifies them in an orderly manner. It provides a practical conceptual framework to better analyze the factors that make up the implementation obstacles of the RTR and their relationships in China's context.

### Objective

This study attempts to explore the multiple obstacles to the implementation of RTR in China through the Van Meter and Van Horn policy implementation approach. Semi-structured and focus group interviews were used to collect data to understand which factors played a role in preventing RTR. This research can make up for the current knowledge gap.

## Materials and methods

### Design

We conducted a qualitative phenomenological study (29) to describe the feelings and experiences of village cadres and villagers on RTR. The phenomenological approach attempts to capture the common characteristics and the meaning of complex phenomena through an intensive study of personal experience (29). Therefore, this method is helpful for an in-depth examination of the practical process of RTR, and exploration of different barriers to RTR implementation and the reasons for failure. The study was conducted in three villages in Jiaozuo City through semi-structured and focus group interviews with seven village cadres and 39 villagers. Thematic analysis is used to analyze data. The report results conform to the Consolidated Criteria for Reporting Qualitative Study (COREQ) checklist (30).

### Sample selection and participation

**Figure 2:**
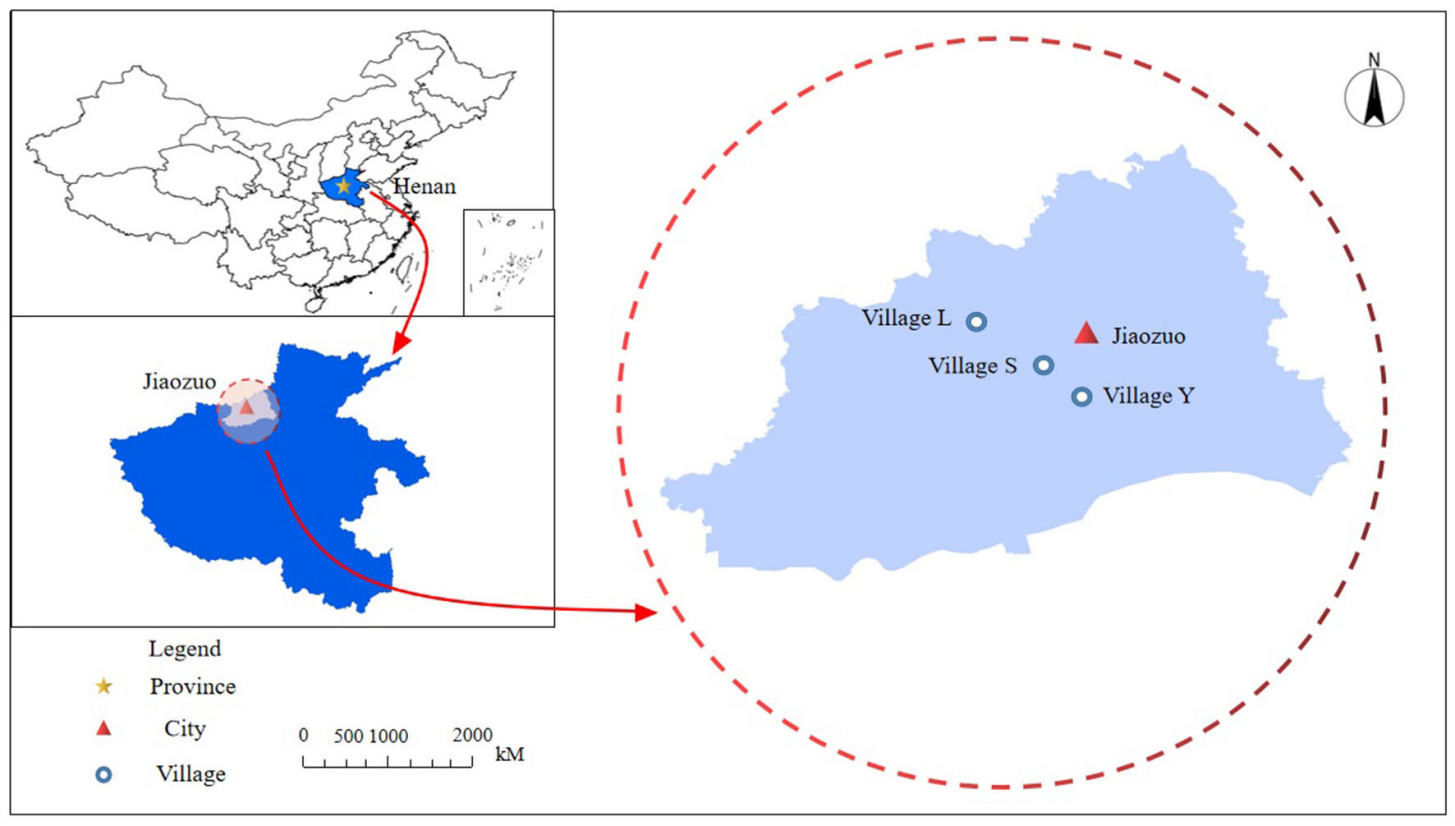
Location of the target areas: Village S, Village Y, Village L, China.

We chose to conduct the research in Jiaozuo City, China. Jiaozuo is located in central China, bordering the Taihang Mountain Range in the north and the Yellow River in the south (Figure 2). It is a central regional city in the city cluster of the Central Plains. According to the principle of case selection, Jiaozuo city was chosen for three reasons: first, external validity. Under China's top-down authoritarian regime, there are many similarities among regions in implementing RTR. Jiaozuo city well reflects these typical characteristics and conforms to the representation. Second, internal validity. We found that Jiaozuo city was rated as an “advanced city in three-year action of Henan Province's rural living environment improvement,” and its jurisdiction of Mengzhou City is one of the nine examples of the national promotion of RTR. It conforms to the principle of the minimum possible crucial case, and can exclude some not crucial variables as much as possible to improve the internal validity of the case. Third, viable cases. The authors are familiar with local dialects and customs, making it easier to collect data. Moreover, the issues of water resources, geographical environment and other aspects in Jiaozuo city are more prominent, which helps us to investigate clearly.

**Figure 3:**
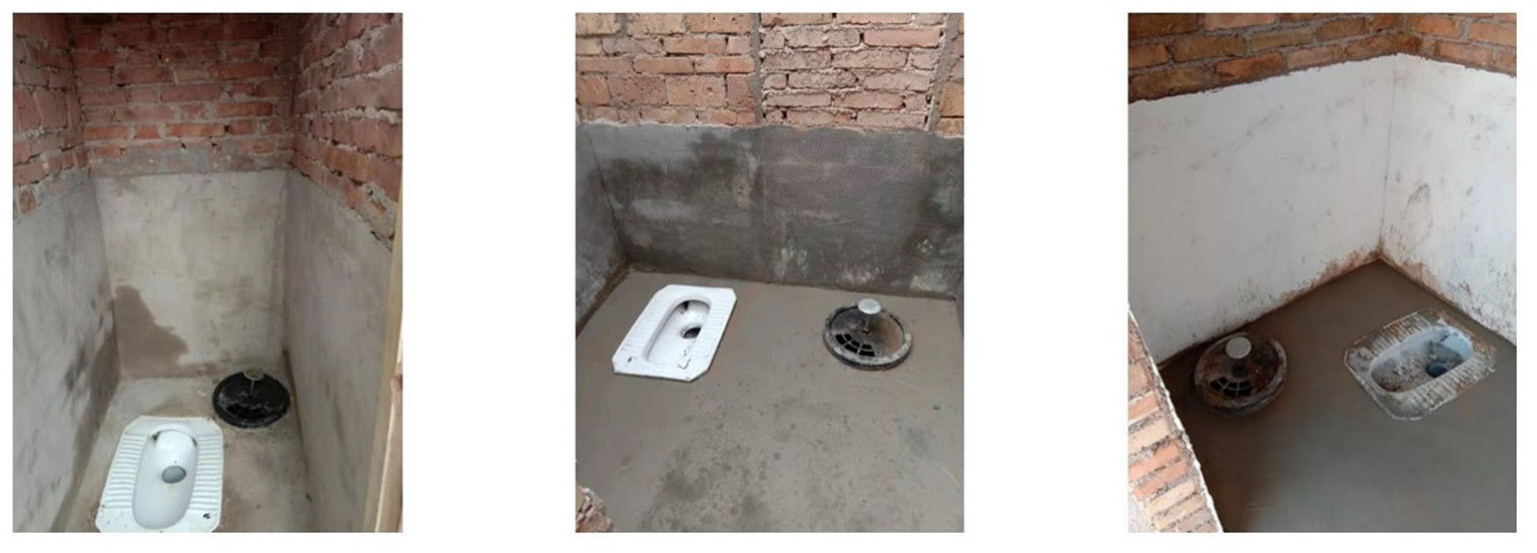
Examples of sanitation; photos provided by survey respondents.

In order to avoid selection bias in one single village, we selected three villages for investigation through the Bureau of Agriculture and Rural Affairs information. The three villages took into full consideration the village size (i.e., number of households), distance from an urban area, sources of fresh water, water fees, the sewer system, the mode of production, and the degree of hollowing out, and other variables. There are apparent differences in Village S, Village Y, and Village L, which can fully represent three different village types in Jiaozuo City and help us obtain in-depth and comprehensive information. Their specific differences are shown in Table 1. The type of new toilets in these three villages is all three-compartment septic tank (Figure 3). The villagers had no other choice. Professional developers recommended by village cadres are responsible for retrofitting toilets.

**Table 1:** Descriptions of the three villages in the study.

**Characteristics**	**Village S**	**Village Y**	**Village L**
Number of households	About 180	About 400	About 200
Distance from urban areas	Nearest, 15 min by bus	Medium distance, 40 min by bus	Farthest, 80 min by bus
Sources of fresh water	Well	Well	Water tower
Water fee	No	CNY 0.5 /ton	CNY 1.5 /ton
Sewer system	Yes	No	No
Mode of production	Agriculture and industry	Agriculture	Agriculture
Degree of hollowing out	Slight	Normal	Normal

Participants are selected according to different principles. Among the cadres, we interviewed all of the village committee cadres who were fully involved in the RTR, and their basic characteristics are shown in Table 2. Qualitative studies collect data from small samples, with different samples pursuing representativeness of abstract types rather than statistical levels. That is, to understand a large number of similar units through an in-depth and detailed analysis of a single research unit. In order to achieve this goal, the participating villagers were recruited through purposive sampling to improve the rigor of the qualitative study (31). Purposive sampling refers to selecting the research object that can provide the most information according to the research purpose, to obtain an in-depth and detailed explanatory understanding of its internal experience. The sampling process fully considered specific criteria related to our research objectives. The participants had to: (a) be a registered rural citizen in the village; (b) have lived in the village for a long time (> 5 years); (c) understand the RTR and be the primary decision-maker of (not) retrofitting toilets; (d) no communication barrier with the researchers; and (e) give informed consent and voluntary participation. At the same time, we fully considered the diversity of the participant's age, gender, family conditions, and has (not) a retrofitted rural toilet to balance the sample. The villagers' basic characteristics are shown in Table 3. The sample size was driven by the concept of data saturation, the time point of data saturation was set when the information provided by interviewees started to repeat with no new themes appearing (32). We interviewed seven village cadres and 39 villagers, none of whom withdrew midway.

**Table 2:** Characteristics of village cadres interviewed (*n* = 7).

**Characteristics**	***N* (%)**
**Age, year**	
30–39	1 (14.28)
40–49	3 (42.86)
50–59	3 (42.86)
**Gender**	
Male	7 (100)
Female	0
**Position**	
Village secretary	2 (28.57)
Village director	3 (42.86)
Village accountant	2 (28.57)

**Table 3:** Characteristics of villagers interviewed (*n* = 39).

**Characteristics**	***N* (%)**
**Age, year**	
20–29	1 (2.56)
30–39	7 (17.95)
40–49	14 (35.90)
50–59	8 (20.51)
60–69	5 (12.82)
70–79	3 (7.69)
80–89	1 (2.56)
**Gender**	
Male	18 (46.15)
Female	21 (53.85)
**Rural toilet retrofitting (RTR)**	
Yes	23 (58.97)
No	16 (41.03)

### Data collection

The methods of semi-structured interviews and focus groups were used to collect data. For village cadres, ten one-to-one semi-structured interviews (including three supplementary interviews) were conducted by the fourth author (LL). Semi-structured interviews can capture participants' experiences and the meaning behind them by asking open questions (30). For villagers, we first conducted 30 one-to-one semi-structured interviews (including three supplementary interviews), completed by the second author (YZ). Meanwhile, five additional focus group interviews were conducted to help absorb and compare the views of different groups in the village, especially different views from different families, those with received RTR and those without. Focus group encourages participants to interact with each other to explore and clarify both individual and shared perspectives (30). At the same time, these focus groups allowed for a triangulation of the themes of the interviews. The focus group interviews were co-conducted by two researchers (YZ and LL) acting as moderator and observer, respectively. Both researchers were well trained in qualitative data collection and had extensive fieldwork experience. We contacted the participants in advance and explained the purpose, methods, and confidentiality principles of the research. After obtaining their consent, we recorded the interviews by on-site recording and taking notes. In order to encourage the free expression of the participants, all the interviews were conducted in an independent area, and no compensation was provided in this study.

In accord with the research purpose, the researchers compiled the interview guide jointly. The interview guide was drafted based on our research questions, literature review and conceptual framework, and was fully discussed by the research team. The interview guide consisted of open questions focusing on the subjective experience. Examples of questions are as follows: “How was your experience with RTR?” “What difficulties did you have in the RTR?” In addition to the reference the interview guide, the interviewers also encourage respondents to fully express their ideas by asking, “Tell us more,” and “Can you explain further?” to obtain more information. The guides' content varied according to the different participants; see Table 4 for details.

**Table 4:** The interview guides.

**Pre-established categories**	**Questions**	
	**Cadres**	**Villagers**
Introductory questions	Please give us a brief introduction to your village.	Please give us a brief introduction to your family.
Key questions	1. Please review the complete process of the RTR. 2. How did you publicize the RTR at the beginning? 3. How did you implement the RTR? 4. How was the completion of the RTR? 5. How did the superior government evaluate the RTR? 6. How do you carry out the maintenance and management of the RTR now? 7. What are the difficulties in implementing the RTR? Why? (1) What are the difficulties caused by the policy standards and objectives? Why? (2) What are the difficulties caused by policy resources Why? (3) What are the difficulties in inter-organizational communication and enforcement activities? Why? (4) What are the difficulties posed by the characteristics of the implementing agencies? Why? (5) What are the difficulties posed by the conditions for policy implementation? Why? (6) What are the difficulties posed by the disposition of policy implementers? Why?	1. Have you ever renovated the toilet? 2. What are the reasons for (not) having the retrofitting? 3. (Renovated) How do you feel about the changes after using the renovated toilet? How is your satisfaction? 4. (Not renovated) Do you plan to renovate the toilet now? Why? 5. What do you think is the main problem of the RTR? Why? (1) What are the difficulties caused by the policy standards and objectives? Why? (2) What are the difficulties caused by policy resources? Why? (3) What are the difficulties in inter-organizational communication and enforcement activities? Why? (4) What are the difficulties posed by the characteristics of the implementing agencies? Why? (5) What are the difficulties posed by the conditions for policy implementation? Why? (6) What are the difficulties posed by the disposition of policy implementers? Why?
Ending questions	Is there anything missing or additional aspects to the topic we are discussing today?	

At the same time, we address potential bias through different data collection methods. With the help of cadres, the researcher (LL) extensively reviewed a series of provincial, municipal, and district-level planning briefs, policy texts and other archives related to RTR to supplement field data. Moreover, researchers (YZ, LL) made field observations of the three villages for ~2 months.

### Data analysis

The interviews were recorded and transcribed verbatim by professional transcription services. The transcriptions were checked and verified for accuracy by the first author (YW) and the third author (CQ), respectively, and treated anonymously. The first author (YW) selectively translated some information (the transcripts quoted herein) into English to present the research results best. We used the thematic analysis method to analyze raw data. Data analysis comprised three steps: (a) we repeated reading the data, and extracted meaningful statements to generate primary coding (such as “unreasonable targets,” “less funds,” etc.,); (b) we further integrated and analyzed the results of primary coding, and formed consistent themes (such as “unreasonable standards and goals,” “insufficient resources,” etc.,) for thinking mapping, and (c) we returned the analytic results to the respondents for verification and confirmation. All researchers analyzed data independently, with disagreeing or vague primary coding and themes discussed many times in research team meetings. This process was iterated in parallel with sampling and data collection to improve validity. Because this study used the Van Meter and Van Horn top-down policy implementation approach as its conceptual framework, the data collection and analysis are more structured than many other qualitative studies (33, 34). Dataset management was performed using NVivo 12.

### Rigor

To ensure the trustworthiness of this study, four criteria specified by Lincoln and Guba are used: credibility, transferability, dependability, and conformability (35). First, researchers follow standardized procedures to complete each step, and record accurate interviews and detailed reflective diaries to improve the credibility of the research. Second, transferability is achieved through a clear and detailed description of the research process. Third, to ensure dependability, on the one hand, we explore commonalities and differences in different individual experiences by different methods. On the other hand, it is independently coded by each researcher and fully discussed to reach an agreement. Finally, we provided rich quotations from the participants to illustrate themes, to ensure the confirmability of the results.

### Ethical considerations

During the entire process, this study strictly followed social science research ethics. Before the interviews, the participants' opinions were fully respected and the purpose and use of the interviews, as well as the requirement for recording, were specified. For confidentiality, all identifying information was anonymous during transcription and translation. The sensitive materials of interviewees involved in the interview process were also processed technically. This study received ethical approval from the Academic Committee of the School of Philosophy and Sociology, Jilin University.

## Results

The data collection process took place between October 2021 and January 2022. The findings of this paper are part of a larger study on “the implementation effect of toilet retrofitting in China,” focusing only on the barriers to the implementation of RAR. Through data analysis, we divided the factors of RTR implementation barriers into six subthemes: (a) unreasonable standards and objectives, (b) lack of resources, (c) uncoordinated inter-organizational communication and enforcement activities, (d) weak and inadequate, unenthusiastic implementing agencies, (e) inappropriate conditions, and (f) passive acceptance by the implementers.

### Unreasonable standards and objectives

It is found that the rationality of policy text and the importance of clear standards and objectives are the premise of effective policy implementation. Through the collection of policy text data, it can be seen that the provincial government, following the *Three-year Action Plan for Rural Living Environment Improvement (2018)* of the central government, formulates the overall plan for the RTR in its province, and clarifies the objectives and tasks, the objects to be implemented, the subsidy standards, the fund-raising, the project management, and other contents. Municipal, county, and township governments should make detailed implementation rules based on the facts of the local reality. However, according to village cadres, the implementation rules in different villages almost wholly copied the implementation rules of the superior. They failed to incorporate local conditions, resulting in a significant waste of resources. Participants stated:

*The policy is good. However, it doesn*'*t make much sense in the countryside. One is that the countryside does not have a sewer system, so it has to be pumped by suction trucks. The cesspool was also small and filled up quickly, and every half a month or a month, I had to pump. It*'*s very troublesome, and it*'*s too dirty. (Interviewee 6, village director, male, age 49)*

*It started with plastic buckets at first, which was what the government asked us to visit and learn from other areas. We did it and found the plastic bucket was leaking. Then we dug the bucket out and changed it into a brick pool. We dug up more than 150. That*'*s too terrible. (Interviewee 4, village director, male, age 45)*

When setting goals, the governments at all levels usually adopt quantitative methods, and some figures are used as the basis for rewards and/or punishments for the implementers. A village director showed the researchers the *Implementation Plan for Rural Toilets and Public Toilets in Jiaozuo (2018)*, which said that “85% of the rural households should be renovated to the harmless sanitary toilet by the end of 2020”. Village cadre interviewees recalled that in 2019, the village committees were notified that they had to carry out the work of the RTR, including the preliminary preparation, the publicity, the allocation of funds, the construction and its supervision. In just a few months, they had to complete the annual retrofitting rate of more than 60% in 2019 and 85% in 2020. Under that extreme pressure to accomplish those tasks, the village cadres, as politically rational people, only paid attention to the final retrofitting rate. However, a high retrofitting rate did not mean improvement in actual quality; most of the time, it was just a figure. One participating cadre stated:

*Our superior is urgently concerned with this. Every meeting, we will be asked about the speed and the number of the RTR……At first, we were asked to renovate 60%, and then stop. Later, we were asked to renovate to 85%, so we started again. We were mobilized every day, and we just wanted to meet the objectives. (Interviewee 4, village director, male, age 45)*

### Lack of resources

The barriers to policy implementation resources include two sub-themes: lack of capital resources and lack of human resources.

In the process of the RTR projects, lack of funds was the most mentioned obstacle. According to the villagers, “*the cost of building a harmless sanitary toilet (three-septic-tank toilet) was roughly CNY 3,000-4,000” (Interviewee 27, villager, female, age 52)*, which is not a tiny expense for rural households. Therefore, to encourage the villagers' enthusiasm to renovate their toilets, the county government would subsidize CNY 500, and the township government would subsidize CNY 300 per household. However, financial difficulties led some families to refuse still to renovate their toilets even with what. The families who underwent toilet retrofitting also complained that they did not receive the final financial subsidy, “*I just received CNY 300, and some even didn*'*t receive CNY 300. They must have taken our subsidy and used it elsewhere to fill up some losses” (Interviewee 43, villager, female, age 47)*. For this, the director in Village L explained, “*We didn*'*t get the county government*'*s subsidy, because we didn*'*t meet tasks of 60%* retrofitting *rate” (Interviewee 6, village director, male, age 49)*. However, the villagers who didn't get the money felt “cheated,” and the lack of payment also dampened the villagers' follow-up enthusiasm for the RTR.

Furthermore, there were inadequate human resources for RTR implementation. As a vast project covering an entire village, the RTR lasted for a long time, and the workload was enormous. In our investigation, we found that the village committee had served as the direct executive body of the RTR. The tasks of the village committee were complicated and onerous, and only two to three officers could be assigned to take responsibility for the RTR (they were responsible for their other tasks at the same time). Faced with the lack of staffing, it was often difficult for the village cadres to complete all of the tasks of the RTR, so they took a perfunctory attitude toward that work. One villager stated:

*What the hell is this... The squatting pot was broken with a big hole when the outside wall was dismantled, and workers didn*'*t repair it. When I asked the village committee to do it, they said yes, but took no action. It is the same with several families. (Interviewee 18, villager, female, age 66)*

### Uncoordinated inter-organizational communication and enforcement activities

Inter-organizational communication and enforcement activities involve two sub-themes: the incoordination between policy implementers and policymakers, and the poor communication between policy implementers and target groups.

The connection between policymakers and policy implementers was realized through top-down task setting and assessment mechanisms, and thus lacked effective communication and cooperation. Policymakers formulated a series of rules and regulations, evaluation standards, and incentive and punishment measures to ensure the RTR implementation. However, in terms of the actual work, the participants of village cadres reflect that the system had apparent problems. On the one hand, the assessment mechanism was not sound. The evaluation of the RTR was completed by township governments. Within a fixed period, evaluators confirmed and took photos of the new toilets in rural households, and thus, the evaluation was completed. However, interviewees think that the evaluators did not pay attention to the difficulties of families who had not carried out the RTR or the difficulties in toilet maintenance and management of the families who had carried out the RTR. On the other hand, township governments focused mainly on the results instead of on the specific obstacles of the implementation process. They often adopted the way of accountability instead of a reward. As a result, the village cadres faced severe unequal rights and responsibilities. In our findings, the village cadres had little active communication with the superior government. They focused only on how to cope with inspection, ignoring how to help villagers use the new toilets to improve the sanitary environment. Once cadre reported:

*People who came to check just had a look, took a photo, and registered it, and that*'*s all. For two years, no one cared about the difficulties. We don*'*t have the energy to do it*.

*Interviewer: they*'*ve built the dry toilets again?*

*Yes, some people think the renovated toilet doesn*'*t work, and then rebuild the dry toilets. The evaluators didn*'*t say anything, so we didn*'*t care. (Interviewee 2, village accountant, male, age 44)*

Policy implementers often communicated informally with target groups, asking them to retrofit toilets, in different ways. One communication effort was to mobilize villagers by using good relationships, such as colleagues and relatives, to renovate their toilets first. The other communication approach was to require villagers to renovate their toilets using coercion, and to threaten the low-income families who did not want to renovate their toilets with the cancellation of their qualification for subsistence allowance from the minimum living security policy^2^. In addition, families who did not renovate their toilets within the allotted time were forced to dismantle their old toilets. Either way, the villagers presented a state of passive participation. Even though their toilets had been renovated, the villagers were not really educated about the advantages of three-septic-tank toilets, so they were quite dissatisfied with the policy. Two reported:

*It*'*s not good when using it. At that time, the village director came to our home for persuasion. It would embarrass him to say no. (Interviewee 27, villager, female, age 52)*

*We are forced to have the retrofitting. The village committee told us to dismantle our old toilets and renovate a new toilet, before a deadline. When the time was due, and we hadn*'*t dismantled it, the village committee sent excavators to pull it down. So I had to do it. (Interviewee 34, villager, female, age 85)*

### Weak and inadequate, unenthusiastic implementing agencies

Data analysis shows that the implementing agencies are characterized by small scale, limited capacity of front-line workers, and less political support.

Firstly, the rural village committees are small, and their working capacity is inadequate. As mentioned above, RTR implementation is the responsibility of village committees. The village secretary described, that two to three cadres of the village committee are often required to finish the RTR task of hundreds of villagers, thus facing severe working pressure. At the same time, village cadres are often poorly educated, with a low level of information access and insufficient resources for professional training. Therefore, they think they lack a complete understanding of the policy, and their working ability cannot be fully realized, thus resulting in a large number of inadequate implementations. The ongoing policy implementation fails to meet the villagers' needs, and the results of that failure further aggravate the doubts and dissatisfaction of the villagers toward the policy implementers, ultimately leading to increasing difficulties in implementing the RTR. Excerpts from a cadre and a villager reflect this barrier:

*According to the requirements, the village committee is only responsible for demolition together, not responsible for unified construction (of new toilets). Villagers themselves buy materials, and they renovate their own toilets. If we are responsible for all, we cannot do it. (Interviewee 1, village secretary, male, age 54)*

*We don*'*t want to change it. We are forced to renovate the toilet by the cadres. In the past, the old toilet was built well with the wall pasted with white porcelain pieces. However, such a good toilet was dismantled. Moreover, this new toilet is not good in use. Those cadres must sell them (the materials) to us to make black money. (Interviewee 9, villager, female, age 42)*

Secondly, the village committees obtain little support and lack organizational vitality. With the proposals of the concepts of construction of new countryside (2005) and targeted poverty alleviation (2013), China has shifted the focus of social policies to rural areas and has carried out a series of public service projects. All the participants of village cadres said, in the area of improving a sanitary environment, several projects have been conducted since 2017, including the treatment of domestic sewage, the protection of drinking water sources, the prevention and control of pollution from livestock and poultry breeding, the classification of domestic garbage, and the RTR projects, among others. The continuous implementation of multiple projects in a short time, along with the lack of support, made the village cadres suffer unbearably. Thus, village cadres have a negative attitude toward the RTR, are not motivated to do the work, and suffer from low organizational vitality. Villagers also confirm this barrier:

*There*'*s no way to fully renovate this harmless sanitary toilet (three-septic-tank toilet), and it may have to be renovated again. Every year, there are several new policies, which make people too tired. However, they (village cadres) cannot do it well because they (the policies) don*'*t conform to reality. Year after year, the situation is the same. (Interviewee 32, villager, male, age 68)*

### Inappropriate conditions

The inappropriate conditions include the inappropriate economic conditions, the inappropriate social conditions, and the inappropriate political conditions.

Inappropriate economic conditions. As mentioned above, retrofitting three-septic-tank toilets requires a large number of family funds, which hinders the willingness of some villagers. Moreover, the government's financial support was only for constructing new toilets, with no support for subsequent maintenance and management costs. Those high costs also made many villagers who had renovated their toilets stop using their new toilets and rebuild their old dry latrines. They reported:

*We rebuilt the old toilet. It (a three-septic-tank toilet) is too expensive. I have to pay suction trucks CNY 20 every time. A few days later, I had to do it again. I need to spend a lot of money every year. (Interviewee 35, villager, female, age 36)*

*A large basin of water was used for flushing the toilet. Dozens of tons of water a month is gone. We farmers feel distressed at this. The water in our village is more expensive than that in the city. (Interviewee 41, villager, female, age 65)*

Inappropriate sociocultural conditions. Sociocultural conditions are the modes of thinking, behavior, and values, moral standards, and customs passed down from generation to generation in a social form (36). Many villagers, who may have used a dry latrine until recently, find that compared to the three-septic-tank toilets, the dry latrines save money and water, and store manure, which is an essential source of agricultural fertilizer. Therefore, villagers tend to recognize a dry latrine widely. Furthermore, the absence of strong publicity for the benefits of three-septic-tank toilets has caused the villagers to have a low recognition of the RTR approach and be dissatisfied with it. One villager summarized their feelings:

*No one wanted to renovate it. No one. The government forced us to do. Many families, like XX, moved out to build the old ones. The manure is used directly for grain and vegetables. Isn*'*t it OK? (Interviewee 39, villager, male, age 32)*

Indeed, even though some families have built new toilets for their children in response to the influence of modern lifestyles, those toilets tend to be used only by younger children and grandchildren, and older people still cling to the traditional ways. A villager stated:

*I don*'*t use the new toilet. I usually go to the open defecation. I built this because my son is grown up. When he comes home with his girlfriend, she will look down upon us, if we don*'*t have these. (Interviewee 16, villager, female, age 49)*

Inappropriate political conditions. For unified planning, standardized management, and beautiful roads, local governments have required villagers to demolish the old toilets outside the houses and build new toilets. This decision has caused significant opposition from the villagers. The style of state governance that pursues simplification and standardization has ignored villagers' common needs, leading to great obstacles in implementing the RTR. A village accountant expressed his attitude:

*At that time, the government above asked us to pull down all the toilets and rebuild the new ones. There was too much resistance if all was pulled down. So we only demolished the obvious ones near the road. The villagers are indeed unwilling if everything is dismantled. (Interviewee 2, village accountant, male, age 44)*

### Passive acceptance by the implementers

The final result of policy implementation depends on the disposition of policy implementers. It includes two factors: one is the disapproval of policy, and the other is the passive response of policy implementers.

The uneven nature of social and economic development and the differences in geographic location, natural environment, and customs have added to the difficulty of implementing the RTR successfully under the unified standards. Village cadres described that they are often in a contradictory dilemma when carrying out their work. On the one hand, as grassroots workers, they need to complete the tasks of their superiors one hundred percent. On the other hand, as one of the villagers, they know that the RTR is not entirely consistent and compatible with the actual situation of the village. In their opinion, RTR does not have a comprehensive understanding of rural housing characteristics, toilet use, and other living habits, so the RTR program will only cause labor and financial loss to the villagers, and it will be unable to achieve the desired results. Therefore, it is difficult for the RTR to obtain the policy implementers' deep understanding and recognition from their hearts. That ambivalence can even cause them to have nothing to do with the actual practice. As one cadre stated:

*We are really in a dilemma. Sometimes the government*'*s idea is good, but not practical. The countryside and the city are different—-in the buildings, the toilet with water flushing can be used naturally, but there is no way of doing it in the countryside. There are no sewers, and water freezes in winter, so this kind of toilet is not as convenient as it is in the city. This issue really gives us headaches. If we don*'*t do it, the superior government will urge us. If we do it, the villagers will also complain. (Interviewee 1, village secretary, male, age 54)*

Under such huge pressure, the village cadres are forced to adopt a stance of passive acceptance. They complete or formalistically complete the task, as far as possible according to the minimum requirements, and then they do not deal with the work beyond the assessment scope. In this way, they can finish their task and minimize the villagers' dissatisfaction. Because of the implementers' disapproval and passive acceptance, the good intentions behind the RTR are effectively abandoned, and the program's real value is ignored. As a villager pointed out:

*These policies are all intermittent. When there was strict scrutiny, we covered the old toilet with slate. When it was over, we used it again*.

*Interviewer: The toilet was not dismantled?*

*It was mainly dismantled—-the outer walls of the toilet [are dismantled]. The bottom of the previous dug pool is not sealed, but covered with slate. (Interviewee 45, villagers, male, age 43)*

## Discussion

The RTR plays a vital role in improving rural sanitation, quality of life and public health (37). In 2018, the Chinese government launched the largest RTR. Despite significant efforts, however, the implementation of the RTR has been complex. Even families with renovated toilets have abandoned them and are reusing traditional dry latrines. Using a field survey of three villages in Jiaozuo City, this paper adopted the Van Meter and Van Horn policy implementation approach to explore the barriers to implementing the RTR.

First, unreasonable policy standards and objectives have a negative impact on policy performance. This finding is consistent with the research conclusion proposed by Wang, Liu & Long that the poor operability of policy texts hinders policy implementation (38). Wang & Wang also suggested the importance of clear and reasonable policy objectives through the “ambiguity—conflict” framework (39). This paper further finds that the state of low ambiguity and high conflict established through quantitative and strict assessment makes the policy implementers only pay attention to the numerical goals, resulting in massive conflicts. It will reduce the satisfaction of the target groups and thus affect the effectiveness of RTR (4).

Second, the lack of economic and human resources is an important factor limiting the realization of the RTR's goal. Some researchers pointed out that some towns and rural households are under heavy economic pressure (40). The lack of economic resources makes it difficult for the villagers to afford the cost of RTR and further maintenance and management, which creates vast barriers (41); further rollout of RTR may fail as soon as the government funding ends (42). Besides, this paper further emphasizes the critical role of human resources, which is rarely mentioned separately in related studies.

Third, the incoordination between policy implementers and policymakers and between policy implementers and target groups will impede policy implementation. Some studies believe that performance assessment is a necessary condition for RTR sustainability (43). All the performance assessments positively contribute to improving the efficiency of RTR (42). However, we found that the top-down assessment leads to a lack of effective communication and cooperation between policymakers and policy implementers, and the problem of “active on the top” and “inactive on the bottom” is prominent. Moreover, Liu found that the rural social governance model makes ordinary villagers lose the right to speak in village affairs in rural areas (44). In the actual RTR process, villagers are often seen as passive recipients (45). This makes it difficult to be accepted by target groups.

Fourth, the final implementer of the RTR is the village committee, which is small in scale, heavy in the task, has few professional training resources, has insufficient working ability, and suffers from low motivation, so it has not been able to do so to improve the policy performance. Our study has confirmed Liu's finding that the village committee is weak and lax, which blocks the RTR implementation (46). Furthermore, a limited number of executors will delay the overall progress of RTR (47). In this paper, we use Van Meter and Van Horn policy implementation approach to refine further the barriers generated by the village committee, and to put forward more targeted suggestions.

Fifth, the inappropriate economic, sociocultural, and political conditions have brought significant obstacles to the RTR. Many scholars have noticed that the unfavorable economic environment negatively impacts on the RTR (38, 46, 48, 49). In social conditions, different countries are constrained by different social norms. For example, India's toilet retrofitting has faced the barriers of the caste system, religious beliefs, and other issues (50, 51). The political environment also affects policy performance. Our research has found that the lens of a clear, simplified and standardized state proposed by James C. Scott is deeply reflected in the RTR implementation process in China (52).

Finally, the unreasonable policy approach of the RTR has made it challenging to obtain the heartfelt recognition of the policy implementers. In their dilemma, they have chosen a form of passive acceptance. Yu found that the government has adopted a series of policies and measures to improve the rural living environment, but the grass-roots government has not actively responded (17). In the face of accountability pressure, the implementers are in a dilemma and choose to passively complete the task, ignoring the projects' sustainability. That approach meets the formalized assessment requirements, but policymakers, policy implementers, and target groups all have spent a large amount of money and time on the RTR, without effectively improving the public health environment.

Fruitful interactions among stakeholders are essential for the success of China's RTR. We have made specific recommendations from the perspective of primary stakeholders, including technology developers, decision makers, executors, and target groups. First, for technology developers, it is recommended to include relevant experts to improve the technical level of the RTR. At the same time, the actual local conditions, including the natural environment and social customs, should be fully considered. Also, as mentioned earlier, local knowledge and the needs of target groups should be addressed in to design of the toilet. Second, for decision makers, we suggest that they should enrich the channels of fundraising to ensure adequate funds, and improve the rational use of those funds to increase enthusiasm for the RTR. At the same time, improving communication channels between decision makers, executors, and target groups is necessary. In addition, we should develop a more reasonable, feasible and stable policy system and supporting facilities, and design scientific evaluation indicators and methods. In the evaluation process, decision makers will need to not only design diversified evaluation indicators, but also conduct follow-up tracking and listen to the executors' and target groups' voices, to ensure that the results of the RTR truly and effectively benefit the target groups. Third, for executors, we suggest that they should increase the staff of the village committee, for better distribution of work tasks. Also, the professional training for executors will need to be strengthened, so that their own working competence and professional quality will allow them to execute their work tasks better. Fourth, for target groups, we will need to establish a working mechanism that ensures the villagers' participation to reduce the work burden of the village committees. In addition, it will also be necessary to increase publicity for the RTR, so that the target groups can fully understand and benefit from the critical role of the harmless sanitary toilets.

### Limitation

Inevitably, this study had limitations, three of which are noteworthy. First, because the RTR was a new policy, there was little information about it in the existing literature. Second, this study was based on field research of three villages in Jiaozuo City. However, China's natural and social environments are relatively complex, and the various provinces and cities have important differences. Therefore, the applicability of the study's conclusions needs further verification. Finally, this study still has insufficient information on the policy decision-making process, which will be an essential aspect to explore to expose all of the different barriers to the RTR. A larger range of qualitative and quantitative studies will need to be conducted in the future to extend the credibility of our existing analyses.

## Conclusions

Using the Van Meter and Van Horn policy implementation approach, the research has found that the barriers to the RTR implementation in China are as follows: unreasonable standards and objectives, lack of resources, uncoordinated inter-organizational communication and enforcement activities, weak and unenthusiastic, inadequate implementing agencies, a list of inappropriate conditions, and an attitude of passive acceptance by the implementers. To solve these barriers, the paper provides specific suggestions from the perspective of primary stakeholders, including technology developers, decision makers, executors, and target groups. Our findings provide valuable knowledge for policymakers as well as policy implementers, which will improve rural sanitation.

## Data availability statement

The raw data supporting the conclusions of this article will be made available by the authors, without undue reservation.

## Ethics statement

The studies involving human participants were reviewed and approved by the Academic Committee of the School of Philosophy and Sociology, Jilin University. The patients/participants provided their written informed consent to participate in this study.

## Author contributions

Conceptualization and methodology: YW and CQ. Software: YZ. Validation and writing—original draft preparation: YW, YZ, and CQ. Formal analysis: YW, YZ, CQ, and LL. Investigation: YZ and LL. Data curation: YW. Supervision and project administration: CQ. All authors have read and agreed to the published version of the manuscript.

## Funding

This work was supported by Special Research Project granted by Northeast Revitalization and Development Institute of Jilin University (Project name: Research on the Path of Community Construction of Party Construction Leading and Five Governance Integration in Jilin Province, grant number: 22dbzx09).

## Conflict of interest

The authors declare that the research was conducted in the absence of any commercial or financial relationships that could be construed as a potential conflict of interest.

## Publisher's note

All claims expressed in this article are solely those of the authors and do not necessarily represent those of their affiliated organizations, or those of the publisher, the editors and the reviewers. Any product that may be evaluated in this article, or claim that may be made by its manufacturer, is not guaranteed or endorsed by the publisher.

## Footnotes

^1^In China, the harmless toilet has six types: three-septic-tank toilet, double-vault funnel toilet, double pit alternate toilet, biogas-linked toilet, urine-feces division toilet and integrated flushing toilet. In this article, the harmless toilet is the three-septic-tank toilet in particular.

^2^Minimum living security policy refers to the social security system in which the government will give certain cash subsidies to the families whose per capita family income is below the minimum living standard announced by the local government to ensure the essential quality of life of the family members.

## References

[1] UNICEF WHO. Progress on Household Drinking Water, Sanitation and Hygiene 2000-2020. (2021). Available online at: https://www.unwater.org/publications/who-unicef-joint-monitoring-program-for-water-supply-sanitation-and-hygiene-jmp-progress-on-household-drinking-water-sanitation-and-hygiene-2000-2020/#:~:text=The%20WHO%2FUNICEF%20Joint%20Monitoring%20Program%20%28JMP%29%20for%20Water,of%20water%20and%20sanitation%20for%20all%20by%202030%E2%80%99 (accessed July 1, 2022).

[2] Katukiza AY Ronteltap M Niwagaba CB Foppen JWA Kansiime F Lens PNL. Sustainable sanitation technology options for urban slums. Biotechnol Adv. (2012) 30:964–78. 10.1016/j.biotechadv.2012.02.007 22361648

[3] Cheng SK Long JY Evans B Zhan Z Li T Chen C. Non-negligible greenhouse gas emissions from non-sewered sanitation systems: a meta-analysis. Environ Res. (2022) 212:1134687. 10.1016/j.envres.2022.113468 35597295 PMC9227720

[4] Cheng SK Zheng L Zhao MY Bai X Li ZF Mang HP. Assessment of two faecal sludge treatment plants in urban areas: case study in Beijing. Int J Agri Biol Eng. (2017)10:237–45. 10.3965/j.ijabe.20171003.3067

[5] Delahoy MJ Wodnik B McAliley L Penakalapati G Swarthout J Freeman MC. Pathogens transmitted in animal feces in low and middle income countries. Int J Hyg Environ Health. (2018) 221:661–76. 10.1016/j.ijheh.2018.03.005 29729998 PMC6013280

[6] Afolabi OOD Sohail M. Microwaving human faecal sludge as a viable sanitation technology option for treatment and value recovery – a critical review. J Environ Manage. (2017) 187:401–15. 10.1016/j.jenvman.2016.10.067 27836558

[7] WHO Water Sanitation Health Team. Water, Sanitation and Hygiene Links to Health: Facts and Figures. (2004). Available online at: https://apps.who.int/iris/bitstream/handle/10665/69489/factsfigures_2004_eng.pdf?sequence=1&isAllowed=y. (accessed March 20, 2022).

[8] Hulton G WHO. Global Costs Benefits of Drinking-water Supply Sanitation Interventions to Reach the MDG Target Universal Coverage. (2012). Available online at: https://apo.who.int/publications/i/item/WHO-HSE-WSH-12.01 (accessed May 5, 2012).

[9] Cheng SK Li Z Uddin SMN Mang H Zhou X Zhang J Zheng L. Toilet revolution in China. J Environ Manage. (2018) 216:347–56. 10.1016/j.jenvman.2017.09.043 28941832 PMC5937855

[10] Tong YD Bu XG Chen C. Impacts of sanitation improvement on reduction of nitrogen discharges entering the environment from human excreta in China. Sci Total Environ. (2017) 9:439–48. 10.1016/j.scitotenv.2017.03.177 28359997

[11] Tang LX Zuo T. Survey and analysis of rural pollution in China – Data from 141 villages in China. China Rural Survey. (2008) 1:31–8.

[12] World Health Organization; Water Sanitation and Health Team. Water, Sanitation and Hygiene Links to Health: Facts and Figures. Geneva: WHO (2004).

[13] Zhang S Li Y Zhang Y Lu ZN Hao Y. Does sanitation infrastructure in rural areas affect migrant workers' health? Empirical evidence from China. Environ Geochem Health. (2020) 42:625–46. 10.1007/s10653-019-00396-2 31428947

[14] Wang DS. Green Book of Rural Living Environments: China Rural Living Environments Development Report (2021). Beijing: Social Sciences Academic Press (2021). p. 58–69.

[15] Shen JB Shi PH. Chinese governance of the “Toilet Revolution”: effectiveness, experience and reflection. Leadership Sci. (2021) 2:41–3. 10.19572/j.cnki.ldkx.2021.02.011

[16] Zhou XQ Prithvi PS Perez-Mercado LF Barton MA Lyu Y Guo SM. China should focus beyond access to toilets to tap into the full potential of its Rural Toilet Revolution. Resour Conserv Recy. (2022) 178. 10.1016/j.resconrec.2021.106100

[17] Yu FW. Rural living environment improvement under rural vitalization strategy. Stud Social Chin Charact. (2019) 2:80–5.

[18] Li J Wang YB Cheng PF. How to speed up China's rural “Toilet Revolution”? – Based on the experience and inspiration of typical countries. World Agri. (2020) 10:20–6. 10.13856/j.cn11-1097/s.2020.10.003

[19] Zhang M. Disparty Analysis of Household Latrine Improvement in Rural China. Master dissertation. Chinese Center for Disease Control and Prevention, Beijing, China (2018).

[20] Luo Q Zhang M Yao W Fu Y Wei H Tao Y. A spatio-temporal pattern and socio-economic factors analysis of improved sanitation in China, 2006–2015. Int J Environ Res Public Health. (2018) 15:2510. 10.3390/ijerph15112510 30423966 PMC6266269

[21] Huang L Qiu M Zhou M. Correlation between general health knowledge and sanitation improvements: evidence from rural China. Npj Clean Water. (2021) 4:1–7. 10.1038/s41545-021-00111-8

[22] Wu S Fu H Li H Ding C Wang M. Residents' willingness to invest in sanitation: evidence from rural China. Desalination Water Treat. (2020) 182:405–13. 10.5004/dwt.2020.25317

[23] Josphat M Kimathi G. Lessons Learnt from Implementation of Outcome Linked Community Led Total Sanitation Intervention in Busia Kenya. Paper presented at the 40th WEDC International Conference, Loughborough, United Kingdom (2017).

[24] Wu SM Zhang Y He BJ. Public willingness to pay for and participate in sanitation infrastructure improvement in western China's rural areas. Front Public Health. (2022) 9:788922. 10.3389/fpubh.2021.788922 35071170 PMC8774769

[25] Zhu L Zhao ZH Wang YP Huang QW Sun Y Bi DP. Weighting of toilet assessment scheme in China implementing analytic hierarchy process. J Environ Manag. (2021) 283:111992. 10.1016/j.jenvman.2021.111992 33486197

[26] Zhou WL Gu YW. Wang XL. Access to sanitary toilets and health outcomes: A panel data analysis using two-way fixed effects model. Math Biosci Eng. (2021) 18:8815–30. 10.3934/mbe.2021435 34814324

[27] Jiang SH. Deconstruction and reconstruction: institutional obstacles and institutionalization strategies of rural “Toilet Revolution” – An analytical perspective of governance. J Party Sch CPC Ningbo. (2019) 41:119–27.

[28] Van Meter DS Van Horn CE. The policy implementation process: a conceptual framework. Adm Soc. (1975) 6:445–88. 10.1177/009539977500600404

[29] Starks H Trinidad SB. Choose your method: a comparison of phenomenology, discourse analysis, and grounded theory. Qual Health Res. (2007) 17:72–80. 10.1177/1049732307307031 18000076

[30] Tong A Sainsbury P Craig J. Consolidated criteria for reporting qualitative research (COREQ): a 32-item checklist for interviews and focus groups. Int J Qual Health Care. (2007) 19:349–57. 10.1093/intqhc/mzm042 17872937

[31] Patton MQ. Qualitative Research & Evaluation Methods: Integrating Theory and Practice. 4th ed. Thousand Oaks, CA: Sage (2015).

[32] Saunders B Sim J Kingstone T Baker S Waterfield J Bartlam B. Saturation in qualitative research: exploring its conceptualization and operationalization. Qual Quantity. (2018) 52:1893–907. 10.1007/s11135-017-0574-8 29937585 PMC5993836

[33] Pope C Ziebland S Mays N. Qualitative research in health care. Analysing qualitative data. BMJ. (2000*)* 320: 114–6. 10.1136/bmj.320.7227.114 10625273 PMC1117368

[34] Kelly M. The role of theory in qualitative health research. Fam Pract. (2010) 27:285–90. 10.1093/fampra/cmp077 19875746

[35] Lincoln YS Guba EG. Naturalistic Inquiry. Newbury Park, CA: Sage (1985).

[36] Wang ZJ. Cultural environment and its influence on human beings. J Beijing Normal Univ. (1992) 1:102–5.

[37] Yan R Cheng SK Chen JG Li XK Sharma S. Operating status of public toilets in the Hutong neighborhoods of Beijing: an empirical study. J Environ Manag. (2021) 287:112252. 10.1016/j.jenvman.2021.112252 33714043 PMC8075803

[38] Wang YS Liu YS Long HL. Regional characteristics and pathway optimization of China′s rural toilet improvement. Journal of Agricultural Resources and Environment. (2019) 36:553–60. 10.13254/j.jare.2019.0245

[39] Wang F Wang R. How do Chinese local governments implement ambiguous policy? – A case study of the “Toilet Revolution” policy in the City A. J Public Manag. (2021) 18:10–21. 10.16149/j.cnki.23-1523.20210818.003

[40] Bai L. Study on cooperative governance path of rural “toilet revolution”. Party & Governance Forum. (2020) 6:33–5.

[41] Wu BC. Improvement of human settlement environment in Qinghai agricultural and pastoral areas under the background of rural revitalization: Effectiveness, challenge and countermeasures. Qinghai Soc Sci. (2021) 4:77–85. 10.14154/j.cnki.qss.2021.04.011

[42] Li Y Cheng SK Li ZY Song HQ Guo M. Using system dynamics to assess the complexity of rural toilet retrofitting: Case study in eastern China. J Environ Manag. (2021) 280:111655. 10.1016/j.jenvman.2020.111655 33309109 PMC7816123

[43] Xue MQ Ma CF. Sustainability of rural toilet revolution projects and their practical logic. Rural Economy. (2019) 9:118–24.

[44] Liu YW. How to deepen rural “Toilet Revolution” from the perspective of innovative diffusion. Chongqing Soc Sci. (2020) 11:119–31. 10.19631/j.cnki.css.2020.011.010

[45] Cherunya PC Ahlborg H Truffer B. Anchoring innovations in oscillating domestic spaces: why sanitation service offerings fail in informal settlements. Res Pol. (2020) 49:103841. 10.1016/j.respol.2019.103841

[46] Liu B. Rural “Toilet Revolution” under perspective of governance theory. J Northwest A F Univ. (2019) 19:28–34. 10.13968/j.cnki.1009-9107.2019.02.04

[47] Roubik H Mazancova J Rydval J Kvasnicka R. Uncovering the dynamic complexity of the development of small–scale biogas technology through causal loops. Renew Ener. (2020) 149:235–43. 10.1016/j.renene.2019.12.019

[48] Li Y Cheng SK Cui JS Gao MJ Li ZF. Mining of the association rules between socio-economic development indicators and rural harmless sanitary toilet penetration rate to inform sanitation improvement in China. Front Environ Sci. (2022) 10:817655. 10.3389/fenvs.2022.817655

[49] Chandana N Rao B. A critical review on sludge management from onsite sanitation systems: a knowledge to be revised in the current situation. Environ Res. (2022) 203:111812. 10.1016/j.envres.2021.111812 34363803

[50] Jacob S Natrajan B Ajay TG. Why don't they use the toilet built for them?: Explaining toilet use in Chhattisgarh, central India. Contribut Indian Sociol. (2021) 55:89–115. 10.1177/0069966720972565

[51] Coffey D Spears D Vyas S. Switching to sanitation: understanding latrine adoption in a representative panel of rural Indian households. Soc Sci Med. (2017) 188:41–50. 10.1016/j.socscimed.2017.07.001 28715752 PMC5641475

[52] Scott JC. Seeing Like a State: How Certain Schemes to Improve the Human Condition Have Failed. New Haven, CA: Yale University Press (1998).

